# Time to start of tuberculosis treatment in penitentiary system of Kyrgyz Republic: A retrospective cohort study

**DOI:** 10.1371/journal.pone.0264252

**Published:** 2022-03-09

**Authors:** Nazgul Soltobekova, Turatbek Kozukeev, Ghirmai Yiehdego, Fatah Labib, Arax Hovhannesyan, Rodolfo Rossi

**Affiliations:** 1 Department for Medical and Sanitary Services of the State Service for Execution of Punishment, Bishkek, Kyrgyz Republic; 2 International Committee of the Red Cross, Bishkek, Kyrgyz Republic; 3 National Center of Phthisiology, Bishkek, Kyrgyz Republic; 4 Independent Consultant, Yerevan, Armenia; 5 International Committee of the Red Cross, Geneva, Switzerland; Hamad Medical Corporation, QATAR

## Abstract

**Background:**

Tuberculosis burden among the incarcerated population is generally higher than that of general population. Early diagnosis and prompt initiation of treatment are key strategies to contain disease transmission. The aim of this study was to determine the time to treatment initiation among inmates with new smear or Xpert MTB/RIF positive pulmonary tuberculosis and explore risk factors associated with delayed treatment initiation in prison settings.

**Methods:**

We conducted a retrospective cohort study using routine health care data from prison settings in Kzrgyz Republic on new pulmonary tuberculosis patients confirmed by smear microscopy or GeneXpert MTB/RIF during 2014–2019. We computed delay in start of treatment—days from specimen collection to treatment initiation—for exposure variables. We dichotomized treatment delay using 10-day cut-off point,and used logistic regression to identify factors associated with treatment delay.

**Results:**

Among 406 cases included into analysis, the median delay to treatment initiation was 7 days [IQR: 2–16 days]. Using 10-day cut-off, 189 (46.6%) patients had delayed treatment initiation. Treatment delay was negatively associated with smear positivity [adjusted OR (aOR) = 0.44, 95% CI 0.29–0.68] compared to smear negative patients, while patients with isoniazid resistant (aOR = 2.61, 95%CI 1.49–4.56) and rifampicin resistant tuberculosis (aOR = 4.14, 95%CI 2.56–6.77) had increased delay compared to patients who were sensitive for both rifampicin and isoniazid.

**Conclusion:**

Timely diagnosis and effective treatment remain the cornerstone of TB control program populations in the general and in prison settings in particular. Prison authorities need to address all potential areas of delay in TB diagnosis and treatment to strengthen their TB control efforts so that prisons remain free of TB for detainees, prison staff and visitors. These include improved supply of TB drugs, early detection of TB cases and improved collaboration with the health authorities outside the prison system.

## Introduction

To date tuberculosis (TB) remains the infectious disease accounting for the highest death toll worldwide. Kyrgyzstan is ranked the first among 53 countries of World Health Organization (WHO) European Region with number of new and relapse cases relative to population. According to WHO estimates, TB incidence in Kyrgyzstan in 2018 was 116 (range: 99–134) per 100,000 population, which was about four times higher compared to regional average and double of that of average of 18 high TB priority countries in the region [1]. Kyrgyzstan is one of top 30 countries in the world with a high burden of multidrug-resistant tuberculosis (MDR-TB) [2].

TB control is challenging especially in correctional settings, where majority of inmates come from group of at risk population, e.g. injection drug users, people living with HIV, tobacco users, homeless, or migrants. Underlying high TB burden in this population is compounded by unfavorable prison conditions, including overcrowding, inadequate nutrition, which might trigger activation of latent TB and contribute to disease transmission [3, 4].

Despite notable reduction of TB notification in the past decade, TB burden in penitentiary system of Kyrgyz Republic remains high: in 2018 Kyrgyzstan notified in total 140 new and relapse TB cases in prisons, equivalent to 1,623 per 100,000 population, which was 16 times higher compared to TB notification rate in general population. This was the highest TB notification rate in prisons reported in WHO European Region in 2018 [1].

The main strategies to control TB are early diagnosis and prompt treatment initiation [5]. At individual level delays in TB diagnosis and treatment can worsen illness, prolong suffering and increase the risk of death [6, 7]. On the other hand, delay of TB diagnosis may facilitate transmission of TB to close contacts and spread the disease. On average it is estimated that one TB patient, if left untreated, transmits the disease on overage to 10–15 individuals per year [8]. However, this number goes much higher in prison conditions, because of overcrowding, and poor air exchange. Therefore, prompt detection, isolation and initiation of treatment of TB is of utmost importance in general but in prison settings in particular. This is especially crucial in penitentiary system where the high prevalence of HIV-infected individuals, intravenous drug use, poor nutrition makes prison population more susceptible to contract TB [4, 9, 10].

Time to TB diagnosis and treatment in penitentiary system and related risk factors has not been evaluated in Kyrgyzstan. Between 2014 and 2018 mean annual reduction of TB incidence in Kyrgyzstan was only 2.0%, which is over two times lower compared to regional 5.1% average annual decline for the same period. Such pace of decline would not be sufficient to achieve the End TB Strategy target of 90% reduction by 2035 in TB incidence rate compared to 2015 [11]. Information on patterns of, and factors associated with delay in TB diagnosis and treatment would help guide future strategies of TB control in prisons.

The objective of the study was to assess the treatment delay and investigate factors associated with it among new pulmonary TB patients confirmed by smear microscopy or WHO recommended rapid diagnostics, notified in the penitentiary system from 01 January 2014 to 10 January 2020.

## Materials and methods

### Design

We conducted a retrospective cohort study among new sputum smear or Xpert MTB/RIF confirmed pulmonary TB patients at the start of the treatment identified in penitentiary system of Kyrgyzstan between 01 January 2014 to 10 January 2020.

### Setting

There are in total 18 penitentiary facilities in Kyrgyzstan including one medical penal institution for people with tuberculosis. The average annual prison population has decreased over the last decade and in 2019 was about 10,100 detainees. Kyrgyzstan’s incarceration rate in 2019 was 161 prisoners per 100,000 population, slightly higher compared to the world median of 140 prisoners per 100,000 population [12]. The Medical Department of the State Service of Execution of Punishment (SSEP) of the Kyrgyz Republic is responsible for medical services of detainees, including for TB. TB case detection in prisons is carried out according to the protocol approved by Ministry of Health of Kyrgyz Republic. Penitentiary system implements TB detection by a combination of passive and active case-finding methods using a standard questionnaire and a chest X-ray covering around 98% detainees according to programmatic reports. Active screening is conducted when of detainees enter into the prison system, and then during the annual mass screening. In selected places reporting the highest number of cases, mass screening is conducted twice a year.

Detention facilities hold new detainees in quarantine unit until they complete their screening tests. Detainees with TB symptoms or abnormal chest X-ray tested with WHO approved rapid diagnostics (WRD) for TB as presumptive TB. Passive detection implies identification of cases among detainees presenting with respiratory symptoms through sputum smear microscopy, Xpert MTB/RIF and chest X-ray. In case of RR-TB, the sputum samples are tested by LPA for rapid second-line DST. Before start of treatment, WRD positive samples undergo smear microscopy culture for tuberculosis and phenotypic DST for first- and second-line TB drugs if culture is positive. If initial GeneXpert test result is negative, patients receive broad-spectrum antibiotics and are re-assessed with WRD test if TB symptoms persist; in addition, the sputum is tested for culture. Health personnel enroll Culture positive patients into TB treatment, and further re-assess culture negative patients to take clinical decision on enrollment into TB treatment.

TB treatment in penitentiary system is centralized. Detainees diagnosed with TB are transferred to specialized penitentiary Institution where they receive integrated care for TB, HIV, drug abuse (e.g. harm reduction) and mental health. SSEP in Kyrgyzstan has two tuberculosis laboratories in two places of detention equipped with microscopes and three GeneXpert machines. Culture and drug sensitivity tests are done at Republican Reference laboratory in Bishkek.

### Study population

Study population are all newly diagnosed sputum smear or Gene Xpert MTB/RIF confirmed pulmonary tuberculosis patients registered in Kyrgyzstan penitentiary system in 2014–2019. We excluded culture confirmed cases, clinically diagnosed pulmonary TB, extra-pulmonary TB and patients with previous history of TB.

### Variables, data source

Main outcome of interests was the time to treatment initiation. Considering extensive periodic implementation of active screening interventions in penitentiary system of Kyrgyzstan, we defined time to treatment as the number of days from date of collection of sputum specimen until the date of start of TB treatment.

The following variables were investigated as possible explanatory variables: age at the start of the treatment, sex, year of start of treatment, HIV status, body-mass index (BMI), presence of cavity on chest Xray, illicit drug use, smoking, sputum smear microscopy results, Gene-Xpert results, and drug-resistance profile.

The source of data was the case-based database of TB patients the medical penal institution of the State Service for Execution of Punishment of Kyrgyzstan built on Epi-Info (Epi-Info version 7.2, CDC, Atlanta, USA). The database captured case-based information about TB patients diagnosed in penitentiary system since 2007. Database focal person entered and updated data from paper-based tuberculosis treatment records, and periodically assessed for accuracy and completeness. Before exporting data for analysis, we checked the dataset for inconsistency and completeness and cleaned manually. In case of discrepancies, we consulted the source documents and medical records to correct the electronic database.

Age was categorized into “< 25 years”,”25–34 years”,”35–44 years”,”45–54 years”,”55–64 years” and” > 64 years”, and BMI “<18.5” and”18.5 and above”, to correspond to WHO case definition on adult under-nutrition [13].

We used median and interquartile range (IQR) to describe the time to treatment initiation due to skewed distribution of outcome of interest. To analyze risk factors, we dichotomized the time to treatment initiation using cut-off point of 10 days. We considered this cut-off as a reasonable timepoint for classification of patients with treatment delay accounting WHO quality indicators recommended to assess timeliness of TB treatment initiation in basic TB facilities as well as the results of similar assessment conducted in general population of Kyrgyzstan [14, 15].

### Statistical analysis

We tabulated socio-demographic and clinical characteristics of study population in absolute number and percentage for categories of variables; and computed mean, SD, median, range and interquartile range of delay by category of variables and as a total.

The odds Ratio (OR) as measure of association between risk factors and treatment delay was calculated and Chi2 test was used to assess the statistical significance of deviation of OR from one. Variables of interest associated with delay with level of a p value <0.25 were included in the multivariate logistic regression model using a forward fitting approach starting from the most associated variable. Adjusted ORs, confidence intervals and p-values were calculated from the final multivariate logistic regression model. Sensitivity analyses were performed by changing the point of delay using cut-off of median and third quartile. Statistical significance of the variables was set for two-sided p values <0.05. Data were analyzed using Stata v15.1 (StataCorp, College Station, TX, USA).

We obtained ethical approval for the study from the Institutional Ethical Committee under the Scientific and Production Association "Preventive Medicine" of the Ministry of Health of Kyrgyzstan on 01 Sep 2019. Study registration number 217.

## Results

**Fig 1:**
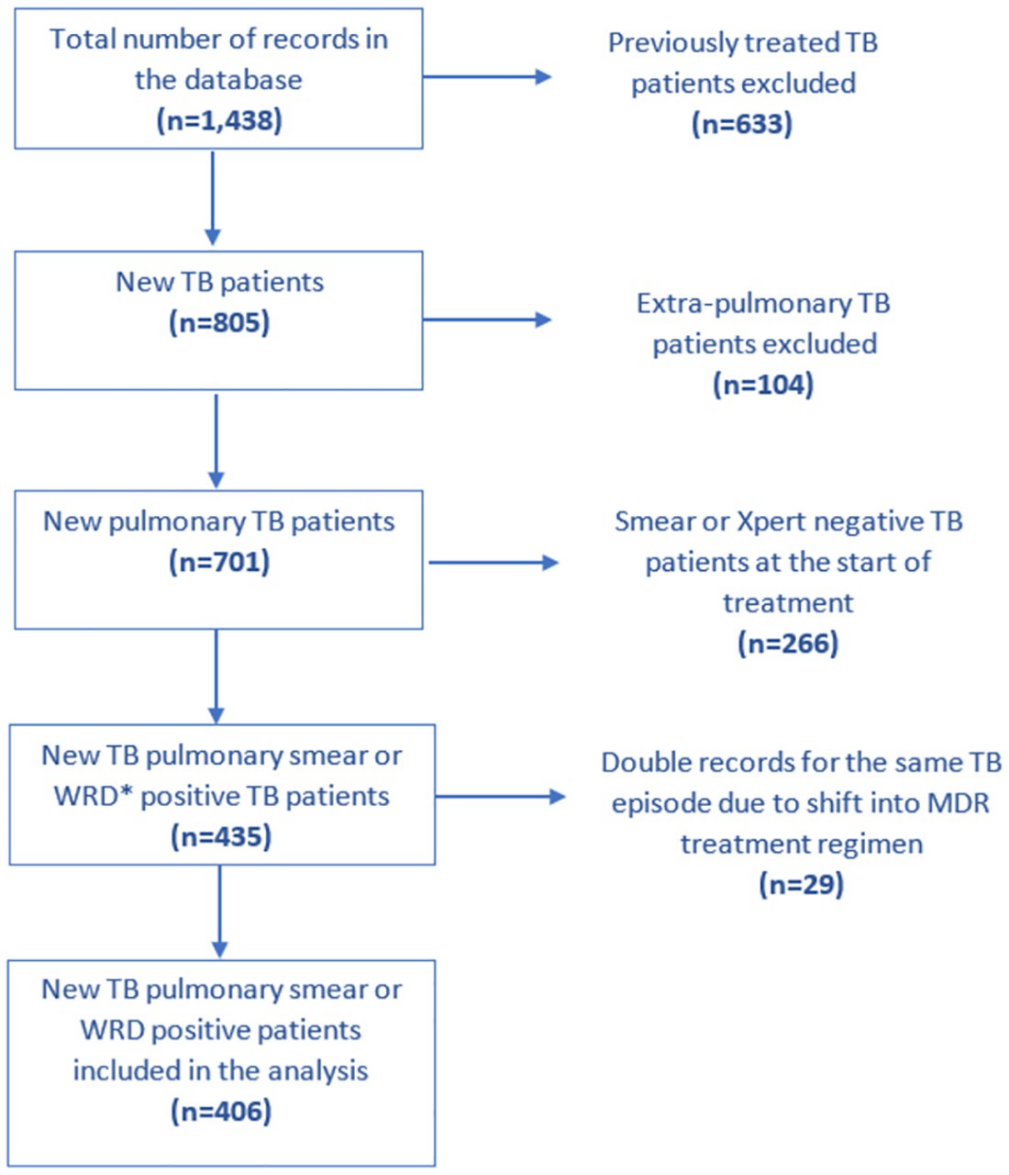
Flowchart of study population based on exclusion criteria. *WRD–WHO recommended rapid diagnostics.

There were 1,438 patients in the dataset. After excluding records that do not meet the inclusion criteria and cleaning for inaccurate and duplicate records, we included 406 new pulmonary sputum smear or Xpert confirmed cases in our analysis as described in Fig 1.

The mean age of study population was 34.7 (SD = 9.8) years, ranging from 18.5 to 71.4 years. Almost all (397; 97.8%) were males, 77 (19.4%) were HIV positive and 27 (6.8%) had lung cavities. Half (202; 49.8%) were smear positive at the start of treatment. About a third of patients had low BMI (119; 32.4%) and 11(3.0%) were overweight. A total of 214 (49.9%) were enrolled into first-line TB treatment. Based on drug-resistance profile to first-line TB drugs at the start of treatment 77 (19.0%) had isoniazid resistance without rifampicin resistance, while 120 (29.6%) had rifampicin resistance with or without isoniazid resistance. Among those with known smoking history, all but two patients were tobacco smokers (98.7%). Moreover, 196 (48.3%) were illicit drug users (See Table 1).

**Table 1:** Time (in days) from the date of specimen collection for microscopy or Xpert MTB/RIF) to TB treatment initiation by demographic and clinical characteristics.

Characteristics	Number	(%)	Time to treatment initiation (day)				
			Mean	(SD)	Median	IQR	Range
**Overall**	406		16.2	37.9	7	(2; 16)	(0–520)
**Age group**							
below 25 years	67	(16.5)	9.6	14.3	5	(1; 12)	(0–80)
25–34 years	155	(38.3)	19.5	39.9	7	(2; 20)	(0–260)
35–44 years	122	(30.1)	14.4	20.7	8	(2; 17)	(0–132)
45–54 years	47	(11.6)	20.0	75.0	6	(1; 17)	(0–520)
over 55 years	14	(3.5)	15.9	19.7	11	(1; 20)	(0–72)
missing 1	1		7.0		7		
**Sex**							
Female	9	(2.2)	8.3	6.6	7	(1;15)	(0–520)
Male	397	(97.8)	16.4	38.3	7	(2;19)	(0–260)
**Year of start of treatment**							
2014–2015	156	(38.4)	19.7	52.5	6	(1; 15)	(0–169)
2016–2017	151	(37.2)	15.7	29.5	8	(2; 18)	(0–260)
2018–2020	99	(24.4)	11.6	13.8	6	(3; 19)	(0–72)
**Body mass index**							
< 18.5 kg/cm^2^	119	(32.4)	22.2	58.9	7	(1; 16)	(0–520)
18.5–24.9 kg/cm^2^	237	(64.6)	14.4	25.9	6	(2; 16)	(0–260)
> = 25.0 kg/cm^2^	11	(3.0)	13.9	12.1	18	(1; 23)	(0–32)
Missing	39		9.6	10.6	8	(1; 12)	(0–47)
**HIV status**							
HIV negative	319	(80.6)	12.9	21.8	6	(2; 16)	(0–260)
HIV positive	77	(19.4)	30.6	73.3	9	(2; 19)	(0–520)
Unknown	10		12.1	15.1	6	(1; 16)	(0–47)
**Cavity**							
None	372	(93.2)	17.0	39.4	7	(2;17)	(0–520)
Cavity	27	(6.8)	6.4	8.5	2	(0; 13)	(0–28)
Missing	7		13.4	11.1	11	(4–20)	(3–35)
**Smear result**							
Negative	204	(50.2)	20.8	46.6	9	(3–20)	(0–520)
Positive	202	(49.8)	11.6	25.6	4	(1–13)	(0–260)
**Resistance to RIF and INH**							
RIF and INH sensitive	209	(51.5)	7.8	10.2	4	(1–11)	(0–62)
INH resistance RIF sensitive	77	(19.0)	15.4	18.9	7	(2–20)	(0–76)
RIF resistance	120	(29.6)	31.4	64.1	13	(5–26)	(0–520)
**Illicit drug user**							
No	210	(51.7)	11.6	19.0	6	(1; 14)	(0–188)
Yes	196	(48.3)	21.2	50.4	8	(2.5; 20)	(0–520)
**Smoking**							
Yes	153	(98.7)	21.2	51.4	9	(2; 20)	(0–520)
No	2	(1.3)	15.5	10.6	16	(8; 23)	(8–23)
Unknown	251		13.2	26.3	6	(0; 13)	(0–260)

SD = standard deviation

IQR = Interquartile range

### Time to treatment Initiation

Median number of days to treatment initiation was 7 days (IQR 2–16). Overall, there was little variation in median time to treatment initiation by categories of variables. Among patients who were sensitive to first-line drugs the median time to start of treatment was 4 days (IQR 1–11), whereas among INH resistant and rifampicin resistant cases the median time to treatment initiation was respectively 7 (IQR 2–20) and 13 (IQR 5–26) days. Table 2 presents the median time to treatment initiation by patient demographic and clinical characteristics.

**Table 2:** Predictors of delay in TB treatment initiation by demographic and clinical characteristics.

Characteristics	Total number	>10 days delays		Univariable			Multivariable		
		Number	(%)	OR	(95% CI)	p-value*	aOR	(95% CI)	p-value**
**Age (every 10 years increment)**				1.02	(1.00–1.04)	0.111	1.01	(1.00–1.04)	0.166
**Sex**									
Female	9	2	(22.2)	1					
Male	397	155	(39.0)	2.24	(0.46–10.97)	0.306			
**Year of start of treatment**									
2014–2015	156	60	(38.5)	1					
2016–2017	151	60	(39.7)	1.05	(0.67–1.67)	0.819			
2018–2020	99	37	(37.4)	0.95	(0.56–1.61)	0.862			
**Body mass index**									
< 18.5 kg/cm^2^	119	49	(41.2)	1.16	(0.74–1.83)	0.509			
18.5–24.9 kg/cm^2^	237	89	(37.6)	1					
> = 25.0 kg/cm^2^	11	6	(54.4)	2.00	(0.59–6.77)	0.258			
Missing	39	13	(33.3)						
**HIV status**									
HIV negative	319	118	(37.0)	1					
HIV positive	77	35	(45.5)	1.42	(0.86–2.35)	0.172			
Unknown	10	4	(40.0)						
**Chest radiological finding**									
No cavity	372	146	(39.2)	1					
Cavity	27	7	(25.9)	0.54	(0.22–1.32)	0.170			
Missing	7	4	(57.1)						
**Sputum Smear**									
Negative	204	95	(46.6)				1		**0.000**
Positive	202	62	(30.7)	0.50	(0.34–0.77)	**0.001**	0.44	(0.28–0.68)	
**Resistance to RIF and INH**									
RIF and INH sensitive	209	54	(25.8)	1			1		**0.000**
INH resistance RIF sensitive	77	35	(45.5)	2.39	(1.37–4.17)	**0.002**	2.57	(1.47–4.50)	
RIF resistance	120	68	(56.7)	3.75	(2.28–6.19)	**0.000**	4.09	(2.50–6.70)	
**Injection drug use**									
No	210	74	(35.2)	1					
Yes	196	83	(42.3)	1.35	(0.90–2.02)	0.142			
**Smoking**									
No	2	1	(50.0)	1					
Yes	153	73	(47.7)	0.91	(0.06–14.99)	0.949			
Missing	168	83	(49.4)	-	-				

*Chi2 p value

**Likelihood ratio test p value

OR = odd ratio

aOR = adjusted odd ratio

CI = confidence interval

### Risk factors of treatment delay

Using a 10-day cut-off point, 189 (46.6%) inmates had delayed treatment initiation. In univariable analysis positive sputum smear result at the start of the treatment was negatively associated, and drug resistance was positively associated with delay while age, year of start of treatment, HIV status, BMI and injection drug use were not. In multivariable analysis, sputum smear and drug resistance remained associated with treatment delay. Smear positive patients had 56% lower odd of delay compared to smear negative patients (aOR = 0.44, 95%CI 0.28–0.68, p < 0.0001). Patients with INH resistance (without RIF resistance) and RIF resistance had respectively 2.6 and 4.1-times elevated likelihood of delay compared to patients who were sensitive to both RIF and INH (Table 2).

We conducted sensitivity analyses dichotomizing treatment delay using the 7-day median or the 17-day third quartile as a cut-off point but the result did not change and only the two variables were associated with treatment delay. Smear microscopy was negatively, and drug resistance profile positively associated with treatment delay while the other variables were not associated (Table 3).

**Table 3:** Significant variables in sensitivity analyses for treatment delays using different cut-off points.

Variables	Cut-off = 10 days		Cut-off = 7 days (median)		Cut-off = 17 days (3rd quartile)	
	aOR*	(95%CI)	aOR*	(95%CI)	aOR*	(95%CI)
**Smear result**						
Negative	1		1		1	
Positive	0.44	(0.29–0.68)	0.39	(0.26–0.60)	0.37	(0.22–0.61)
**Resistance to RIF and INH**						
Sensitive	1		1		1	
INH resistance RIF sen sensensitive	2.61	(1.49–4.56)	1.81	(1.05–3.14)	3.09	(1.63–5.87)
RIF resistance	4.14	(2.56–6.77)	3.76	(2.30–6.13)	4.64	(2.66–8.10)

aOR = adjusted odd ratio

CI = confidence interval

*Adjusted for age, smear result and resistance to RIF and INH

## Discussion

Current study demonstrated that half of the inmates with positive sputum smear and/or Gene Xpert MTB/RIF result were enrolled to TB treatment within seven days after specimen collection, whereas a quarter of the patients started treatment 16 days after the specimen collection. Time to treatment was shorter for sensitive TB patients, compared to patients with resistant forms of tuberculosis. Our study findings are similar to previous study conducted in civilian population in Kyrgyzstan which demonstrated median of 10-day (IQR 6, 16) delay between diagnosis of RR-TB and treatment initiation [15].

Information is scanty on time to start of treatment in prison settings around the world. Compared to two studies from correctional settings of high income countries, median time to treatment initiation in our study was longer than that reported from the jails of United States [16] and in Japan [17]. However, our study showed shorter time to start of treatment compared to the one reported in Iran, which had a median of 30-day (IQR: 18–31) delay among prison population [18].

A recent systematic review and meta-analysis illustrated that in civilian settings in low and middle income countries (LMIC), the median health system delay (defined as the time interval between the patient’s first consultation with a health care provider and the date of diagnosis) ranged from 2 to 128.5 days [IQR 12, 34] [19]. Although the definitions used for in this review differ from our study, several settings reported delay longer than that of our study indicating the challenges faced in health systems in LMIC for early diagnosis.

Because of higher TB prevalence in prison settings, we expect health personnel to suspect and investigate TB more than in general health care settings. Thus, studies that compared treatment delay in civilian and correctional settings have demonstrated that time to treatment in prison is shorter compared to civilian settings [18, 20]. Another nationwide study from Ukraine demonstrated comparable health service delay among incarcerated and civilian population [21].

In correctional facilities of Kyrgyzstan, we identified two clinical characteristics, specifically drug resistance profile and sputum smear result to be associated with delay in the start of treatment. In closed institutions like prisons, where periodic screenings are conducted, the time to start of treatment is heavily affected by the health systems and procedures in place such, such as logistics of transportation of biological materials, the turn-around-time for laboratory results, procedures for transfer of inmates from one prison to the prison TB hospital. Moreover, patient related factors could affect treatment delay. Before starting treatment, patients should sign informed consent for the treatment. In our study, majority of the extreme delay were due to patient’s refusal to start treatment. The hospital houses patients that refuse treatment in special barrack to maintain infection control. Majority of the patients that refuse treatment were asymptomatic and were detected through active TB screening. Refusal to start the treatment also to some extent could be due to TB diagnosis in Kyrgyzstan penitentiary system being linked with exclusion from early release, among those eligible for it.

We found that sputum smear positive patients were more likely to start the treatment earlier compared to sputum smear negative cases as was observed in Croatia and in Taiwan [22, 23]. This is mainly due to sputum smear positive patients were fast-tracked to prevent disease transmission in correctional facilities.

As expected, drug-resistant cases had longer time to treatment initiation compared to drug-sensitive TB. Drug-resistant TB have to go through adherence counselling and health education before starting treatment and give informed consent. In addition, baseline tests are required before deciding on treatment regimen, which may explain the longer delay among DR cases.

As our study used data collected for routine health care with already defined variables, the effect of other potential risk factors for treatment delayed such as mode of detection, social status, and level of education could not be measured leaving a potential residual confounding. Moreover, we analyzed only the time from the sputum collection until the start of treatment. To get a complete picture on delay, patient-related and health-system-related delays need to be investigated. Time from start of symptom until consultation to the health system will comprise patient-related delay and from consultation until the start of treatment will consist the health-system-delay. Our data captured part of the health system delay and our findings are underestimate of the actual health system delay as the delay will be more if we consider the time from consultation to the health facility. However, we do not expect big difference due to the active screening practices in the prison system that detects significant part of the patients even before developing TB symptoms.

Our findings are representative for the entire study population (newly diagnosed TB patients in prison settings) as we excluded only few subjects from the analysis due to missing data on key variables, making selection bias less likely. We included both drug sensitive and drug resistant forms of TB in our analysis; and we could assume that our study results provide a general insight of time to treatment in correctional settings in Kyrgyzstan. We applied different cut-off points of delay, which allowed us to examine the possible alteration of risk factor among those with extreme delay and compare this with the findings with lower cut-off. In addition, our study population was large enough and allowed to assess the secular trend of outcome.

## Conclusion

This is the first study, which evaluated the delay TB treatment initiation in penitentiary system of Kyrgyzstan and one of the largest such study in a prison setting with high MDR and TB/HIV burden. FAST, an acronym that stands for Find cases Actively, Separate safely and Treat effectively has been promoted as a strategy to control tuberculosis infection in congregate settings [24]. The effectiveness of TB case-finding and timely initiation of treatment in prisons could be influenced by several factors. Prisoner’s awareness on TB, stigma related to TB, the availability and quality of diagnostic facilities, competence of prison health care providers, existing coordination and communication with health structures of civilian sector, presence of clear responsibilities of prison staff and commitment of prison authorities.

Majority of prison population come from members of the community with lower socioeconomic status and may be less likely to be aware of TB symptoms, contributing to a delay in care seeking behavior. Once the prisoner recognizes the TB symptoms, accessing health care could be challenging due to inmate-enforced internal rules, tightness of the regime inside the prison and availability of logistic and human resource facilities. When a prisoner reports to the health facility, detection of tuberculosis relies on quality health care service dependent on presence of qualified health personnel and equipped facilities, rare commodities in most prisons in the developing world. Moreover, access to referral centers with advanced diagnostic facilities outside prison settings could be delayed due to several factors including status of the prisoner and availability of security escorts and associated logistics.

Timely diagnosis and effective treatment remain the cornerstone of TB control program populations in the general and in prison settings in particular. Prison authorities need to address all potential areas of delay to ensure that prisons remain free of TB for the detainees, prison staff and visitors. Prison health facilities need to collect routine data on date of onset of TB symptom, date of first report to the health facility for TB symptom, date of laboratory diagnosis and date of start of treatment so that all elements of delay could be analyzed for better program performance. Health education and patient counselling in prisons need to be strengthened to gain patient’s confidence to health recommendations and reduce treatment refusal. Moreover, rules and regulations that discriminate TB patients in prison settings need to be reviewed and avoided to ensure that detainees with TB could report to health facilities without fear negative consequences on their stay in prison. For effective interaction between the State Penitentiary Service and the Ministry of Health in the provision of medical services to detainees, a joint plan of the SSEP and the Ministry of Health of the Kyrgyz Republic has been developed. This includes:

- Systematically carry out health education session in prisons. Raise awareness of TB among prisoners, cough etiquette, basic personal hygiene practices. Promptly seek medical attention if symptoms are reported.
- On admission to prisons, a standardized tuberculosis questionnaire should be used for all prisoners. (cough, night sweats, fever, etc.), if symptoms are present, they should be confirmed with Xpert MTB / RIF.
- Conduct annual mass screening for tuberculosis using questionnaires and X-Ray in all prisons.
- Sputum samples from patients with suspected TB should be sent for Xpert MTB/RIF testing to laboratory in prison within 24 hours. For facilities located in remote areas, refer to the nearest civilian TB center.
- Suspected or confirmed TB cases should be isolated until confirmation or before being transferred to a TB hospital.
- Report to the SSEP TB coordinator
- Transfer the patient to a TB hospital for treatment as soon as possible. (No more than 3 days from the moment of TB detection),
- If a case of tuberculosis is detected in remote prisons (colony 10, pre-trial detention center-3, pre-trial detention center-4 and pre-trial detention center-5), actions should be coordinated with the territorial TB coordinator of the civil sector.

Every patient with tuberculosis should begin treatment for tuberculosis within the first 3 days after diagnosis. To achieve this, the following recommendations are proposed:

- Timely request for TB drugs and always have a monthly buffer stock of TB drugs (at least 5 patients for susceptible TB and 5 patients for DR-TB); this will increase access to TB drugs
- Provide effective patient counseling before starting TB treatment.
- All new TB patients should be started on TB treatment immediately without waiting for DST results.
- Collect standardized information on the date of onset of TB symptoms, the date of the first reporting of TB symptoms to the health facility, the date of collection of the samples, the date of the results, and the date of initiation of treatment so that all elements of the delay can be analyzed to improve the program.
- Improved collaboration with the civil sector TB laboratory where DST is performed. Access to a unified national database for obtaining laboratory results online within a short delay
- Improve communication between prisons, territorial health facilities and health authorities;
- Conduct TB Consilium systematically (internal once a week and external Consilium once a month).
- Prison health workers should be trained on a regular basis (online and offline trainings)

## Supporting information

S1 Dataset (XLSX)
